# Non-Dependent and Dependent Daily Cannabis Users Differ in Mental Health but Not Prospective Memory Ability

**DOI:** 10.3389/fpsyt.2018.00097

**Published:** 2018-03-27

**Authors:** Ruth Braidwood, Samantha Mansell, Jon Waldron, Peter G. Rendell, Sunjeev K. Kamboj, H. Valerie Curran

**Affiliations:** ^1^Clinical Psychopharmacology Unit (CPU), University College London, London, United Kingdom; ^2^Cognition and Emotion Research Centre, Australian Catholic University, Melbourne, VIC, Australia

**Keywords:** prospective memory, cannabis, future event simulation, cannabis dependence, addiction

## Abstract

Research suggests that daily cannabis users have impaired memory for past events, but it is not clear whether they are also impaired in prospective memory (PM) for future events. The present study examined PM in daily cannabis users who were either dependent (*n* = 18) or non-dependent (*n* = 18), and compared them with non-using controls (*n* = 18). The effect of future event simulation (FES) on PM performance was also examined. Participants were matched across groups on age, gender, and highest level of education. The virtual week (VW) was used to objectively assess PM abilities, both at baseline and following FES. Other measures used were: cannabis use variables, immediate and delayed prose recall, phonemic and category fluency, spot-the-word test (premorbid intelligence), Beck Depression Inventory, Beck Anxiety Inventory, and a measure of schizotypy (Oxford-Liverpool Inventory of Feelings and Experiences: unusual experiences subscale). No group differences were found in PM performance on the VW, and FES did not improve PM performance in any group. Dependent cannabis users scored higher on depression, anxiety, and schizotypy than both other groups with non-dependent cannabis users scoring at a similar level to controls. There were no group differences in alcohol use. Findings suggest that when carefully matched on baseline variables, and not differing in premorbid IQ or alcohol use, young, near-daily cannabis users do not differ from non-using controls in PM performance.

## Introduction

Cannabis has consistently been the most commonly consumed illicit drug in the world, with an estimated 120–190 million users worldwide (1). Currently, it is increasingly becoming a legal drug for medical and/or recreational use in many parts of the globe and its use is increasing (1). Rapid changes in the legislation of the drug strengthen the importance of research on the effects of cannabis on mental health and cognitive functioning.

Frequent cannabis use has been associated with depression and anxiety (2). Higher rates of depression and anxiety have been observed in frequent users who are dependent on cannabis compared with frequent users who are non-dependent, who score similarly to the general population (3). There is also an association between use of high-potency cannabis and psychosis (4).

One of the most robust acute effects of cannabis is impairment of memory (5) and frequent, chronic use of the drug has also been associated with significantly poorer memory performance (5, 6). However, the majority of evidence on the effects of cannabis use on memory focuses on retrospective memory (memory for past events). Research into the effects on future-based memory processes remains relatively neglected.

Prospective memory (PM) is a vital aspect of everyday memory and refers to the ability to enact intended actions at an appropriate moment in the future (7). To-be-remembered actions can be event-based (e.g., collecting a prescription when passing a doctor’s surgery), time-based (e.g., meeting a friend at 18:00), or activity-based (e.g., calling your sister after posting a letter). As such, these forms of PM depend, respectively, on whether the cue for PM performance is the appearance of a certain stimulus, the passage of a certain amount of time or the completion of an activity. Actions may also be regular (e.g., taking medication every morning), or irregular, one-off or infrequent actions (e.g., attending a dentist appointment). PM ability relies on retrospective memory to retain knowledge of the intention and the cue, and on executive planning and motivation functions to coordinate intended actions (8).

Impairments in PM performance have been reported among individuals with alcohol dependence (9), methamphetamine users (10), long-term opiate users (11), heavy social drinkers (12), and MDMA users (13). Aside from the adverse consequences of PM deficits on everyday functioning, such deficits may also specifically impair an individual’s ability to apply planned relapse prevention strategies when aiming to curtail substance use. An improved understanding of PM in individuals with substance dependence might thus help to inform treatment delivery during rehabilitation.

There is evidence that deficits in PM can be overcome by planned cognitive strategies such as future event simulation (FES). FES involves “pre-experiencing” future events using structured mental imagery (14) whereby the individual is asked to vividly imagine performing the future action during encoding. The constructive episodic simulation hypothesis proposes that episodic memory combines the details of past experiences (e.g., objects, people, and locations) to depict potential future events (14). There is some evidence that FES may improve PM performance on a widely used, objective measure of PM, the virtual week [VW (15)]. A double-blind placebo-controlled trial found that acute alcohol induced deficits in event-based PM in healthy individuals were overcome by FES (16). Similarly, FES significantly improved the performance of heavy social drinkers on event-based tasks on the VW (12). However, a study comparing VW performance in alcohol-dependent individuals and social drinkers found FES improved time-based PM only for the latter (9).

Studies investigating the effects of cannabis use on PM performance have found that users perform significantly worse than healthy controls on irregular event-based PM tasks (17–22), and on irregular time-based tasks (19–21, 23). However, effect sizes in these studies varied markedly, as did important aspects of the PM task used. There was also large variation in how a “cannabis user” was defined, ranging from minimal use of “some cannabis use in the past year” (17) to “at least four times in the last month” (22). Cannabis users and controls were also generally poorly matched on other drug use (especially of the classic amnesic drug, alcohol) and demographic variables, including educational level. Furthermore, no study to date has investigated whether deficits in cannabis users’ PM performance could be overcome by FES.

The present study therefore sought to determine whether individuals who used cannabis on a frequent basis (≥4 days/week) and fulfilled criteria for dependence or not differed from each other and from a control group on the VW. It also aimed to see if FES improved PM performance in the three groups (control non-users, dependent, and non-dependent daily users). In line with previous research, we hypothesised that cannabis users would exhibit a deficit in irregular event- and time-based PM compared with control participants, with dependent users showing a greater irregular PM deficit than non-dependent users. We also hypothesised that there would be poorer performance on regular PM in cannabis users compared with control participants. As no previous study had used FES with cannabis users, this aspect was exploratory.

Given the role of memory and executive function in PM processes, these domains of cognition were also assessed along with depression, anxiety, and schizotypy (“psychosis-proneness”) as depression and anxiety are higher only in dependent frequent users of cannabis (3).

## Materials and Methods

### Participants and Design

An independent group design was used with 56 participants: 18 dependent cannabis users, 18 non-dependent cannabis users, and 18 non-cannabis using controls. Participants were recruited *via* advertisements at local university campuses and around public areas, and on social media websites. Interested individuals were telephone screened for eligibility. The inclusion requirement for cannabis users was to use at least 4 days per week (i.e., more days than not). Additional information during the telephone screening included age at which participants started using cannabis, number of grams used per week, and score on the five-item severity of dependence scale [SDS; (24)]. We classified users as dependent (score ≥3 on the SDS) or non-dependent (score <3). According to Swift et al. (25), an SDS score of three or above indicates probable cannabis dependence, with sensitivity of 64% and specificity of 82% when compared with the “gold standard” diagnostic criteria for cannabis dependence (DSM-III-IR). All participants were required to have limited other illicit drug use (twice a month or less) and no history of substance dependence. Potential controls were also considered ineligible if they had a history of frequent cannabis use (twice a month or more).

Demographic information was collected for all individuals including age, gender, and highest level of education. We also asked all individuals about their alcohol use and use of other drugs. All participants were required to speak English fluently. Exclusion criteria included: being under 16 years old, a current or historical diagnosis of dependence on any substance other than cannabis or tobacco, weekly alcohol consumption exceeding 21 units for women or 28 units for men, a history of traumatic brain injury or stroke, a current or recent (last 3 weeks) experience of psychosis, in current treatment (psychological therapy or pharmacological) for a mental health problem other than anxiety or depression, a diagnosis of a learning disability, reading difficulties, or current use of antipsychotic medication or benzodiazepines.

All participants verbally agreed to refrain from consuming any illicit drugs and alcohol on the day of the testing session. Participants from each of the three groups (dependent cannabis users, non-dependent cannabis users, and controls) were actively selected to match each other in age, gender, and highest level of education as closely as possible. The study received ethical approval from the University College London (UCL) Research Ethics Committee (5402/001) and all participants provided written informed consent prior to taking part.

### Measures

#### Prospective Memory

The VW (15) is a virtual board game that requires participants to move a counter around a board (displayed on a laptop screen) by rolling an electronic die. Participants work their way around the board, with one circuit of the board representing one virtual “day.” The virtual time of day is shown on a clock in the centre of the board and the time passes as the counter moves around the board. Over the virtual day, there are 10 green “E” squares on the board to pass through. When a participant’s counter falls on or passes an “E” square, they are instructed to click on the event card button on the board. This event card symbolises a time-appropriate event occurring in the virtual day. For example, the first event cards depict morning activities such as eating breakfast, and the last event cards depict evening activities such as eating dinner. Each event card requires the participant to select a multiple-choice answer in response to a given activity, such as what to eat for breakfast. Throughout each virtual day, participants are assigned a number of tasks they must remember to perform at points later in the day (as a measure of PM). In the version of the VW used in this study, each day contained four “time-based” tasks to be performed at specified times of day (as displayed on the central 24-h clock), and four “event-based” tasks to be performed in response to particular events.

#### Episodic Memory

The story recall subtest of the Rivermead Behavioural Memory Test (26) was used as a measure of verbal episodic memory. Participants listen to a short passage of prose and are asked to immediately repeat back everything they can remember. They then repeat the recall task after a delay. Scoring was standard.

#### Executive Function

Three verbal fluency tasks were used: phonemic (words beginning with the letter “g”), category (vegetables), and drug fluency (alcohol-related words). In addition, the two cannabis using groups completed a cannabis-fluency cannabis-related words. Participants had 60 s to name as many words relating to each category as they could. The three main tasks were counterbalanced by topic.

#### Premorbid Intelligence

The spot-the-word test [STWT; (27)], a task requiring participants to select the real word from each of 60 letter-string pairs containing one word and one non-word, was used as an estimate of premorbid intelligence. The STWT has previously demonstrated convergent validity with the Wechsler Adult Intelligence Scale (28).

#### Cannabis Use

**Figure 1:**
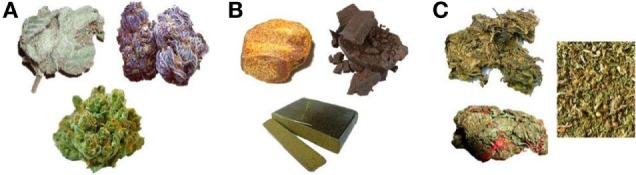
Preparations of cannabis.

Using an in-house tool, participants were asked to select one of three pictures (see Figure 1) that best represents the type of cannabis they use most frequently, and to then estimate the approximate percentage of time they use each of the three types. Each picture shows different preparations of cannabis: high-potency floral preparation which typically contains very high levels of tetrahydrocannabinol (THC) and little or no cannabidiol (CBD), often referred to as “skunk” (Figure 1A), compressed resin or “hash,” typically containing higher levels of CBD and lower levels of THC (Figure 1B) and traditional dried herbal material, referred to as “bush weed” or “Thai weed,” which contains much lower levels of THC but little or no CBD (Figure 1C).

#### Depression, Anxiety, and Schizotypy

The Beck Anxiety Inventory [BAI; (29)] and Beck Depression Inventory [BDI-II; (30)] were used to assess anxiety and depression. The Oxford-Liverpool Inventory of Feelings and Experiences [O-LIFE; (31)] is a measure of schizotypy, or “psychosis-proneness.” The unusual experiences subscale only was used as it has been found to be a reliable and time-efficient scale to explore psychosis-proneness (32).

### Procedure

All participants attended a one-off testing session at the Clinical Psychopharmacology Unit at UCL, which lasted approximately 2.5 h including breaks. Participants were compensated for their time.

Participants from both cannabis groups first completed questions about the type of cannabis they used. All participants were then introduced to the VW. A trial day was completed to orient participants to the task, during which they followed the instructions on-screen and had an opportunity to ask questions. Participants were not permitted to start the VW until they could successfully articulate all of the regular PM tasks to ensure they had encoded the information. They were also asked to read aloud every event card in the game. Participants then completed their first two virtual days. The tester did not provide feedback on accuracy.

**Figure 2:**
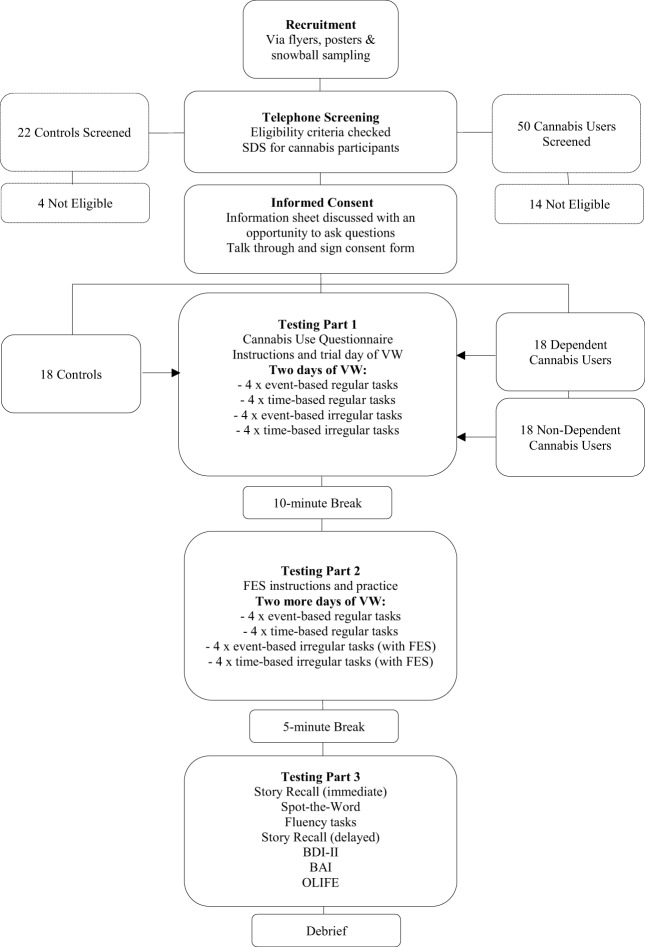
Study flow diagram.

After a 10-min break, participants were instructed on the use of the imagery technique (FES) which they were told to use when forming intentions related to all irregular events presented over the next two virtual days of the VW. This involved imagining oneself performing a task in as much detail as possible, including details like the setting and course of events, the time of day, and the people and objects in the scene. Participants were encouraged to set the imagined event in their own daily life (e.g., imagining themselves shopping in their regular supermarket if given the task of buying groceries). Participants then carried out two more days of the VW, during which they were prompted by the tester to imagine each irregular task for 10-s after the task had been set. As such, 2 days of the VW were completed without FES, followed by 2 days with FES, Participants were then given a 5-min break, after which the remaining tasks were administered. Figure 2 shows the full study protocol. Note, this study was part of a larger study on future thinking in cannabis users. An additional task involved imagining various consummatory behaviours, which will be reported separately.

### Statistical Analyses

Analyses were primarily performed on IBM SPSS Statistics Version 22. A confirmatory Bayesian analysis employed the JASP programme Version 0.8.1.2 (33). There was no missing data. Variables were visually and statistically checked for normality. Means and SDs were reported where parametric statistical tests were employed. Taking sample size into consideration (34), skewness, and kurtosis *Z-*scores of ≥3.29 were used to identify non-normality. Using this criterion, frequency of type of cannabis used as a percentage of all occasions, amount of cannabis (grams per week), and the BAI data violated assumptions of normality. For non-normal variables measured in only two groups (i.e., cannabis-related variables), non-parametric (Mann–Whitney *U* tests) were used to compare groups and central tendency and dispersion were described using medians and inter-quartile ranges. For all other variables, parametric χ^2^ tests were used to compare categorical variables, and *t*-tests (two group comparisons) or ANOVAs (three group comparisons) were used to detect group differences for continuous data.

The assumption of homogeneity of variance (as evidenced by Levene’s test) and normality according to our criteria (*Z* ≤ 3.29) was met for all VW variables. VW data were analysed with repeated measures ANOVAs, with Bonferroni-adjusted pairwise comparisons performed to explore *post hoc* effects. To explore relationships between significant variables, Spearman’s *rho* (*r_s_*) correlations were conducted (on both parametric and non-parametric variables, to allow for direct comparison). Correlations were conducted with an adjusted alpha of 0.01 to minimise Type-I error. Data were also examined for extreme values. One participant’s score in one of the VW variables was four SDs from the mean, and was Winsorised to the next highest non-outlying value.

## Results

### Group Demographics

There were no differences between the groups in terms of gender, χ^2^ (2, *N* = 54) = 1.94, *p* = 0.38, age, *F*(2, 51) = 0.15, *p* = 0.89, highest level of education, χ^2^ (4, *N* = 54) = 2.20, *p* = 0.67, spot-the-word score, *F*(2, 51) = 1.00, *p* = 0.37, or units of alcohol consumed per week, *F*(2, 51) = 0.12, *p* = 0.89 (Table 1).

**Table 1:** Group demographics, alcohol use, and spot-the-word scores across the dependent cannabis, non-dependent cannabis, and control groups.

	Dependent cannabis users (*n* = 18)	Non-dependent cannabis users (*n* = 18)	Controls (*n* = 18)

	*n*(%)	*n*(%)	*n*(%)
Gender			
Male	9 (50)	10 (55.6)	6 (33.3)
Female	9 (50)	8 (44.4)	12 (66.8)
Highest level of education			
GCSE or vocational qualification	1 (5.6)	2 (11.1)	3 (16.7)
A level	8 (44.4)	5 (27.8)	5 (27.8)
University degree	9 (50.0)	11 (61.1)	10 (55.6)

	**M (SD)**	**M (SD)**	**M (SD)**

Age (years)	24.2 (5.1)	23.9 (3.7)	23.4 (3.7)
Spot-the-word score	49.5 (3.5)	47.2 (5.8)	48.6 (5.3)
Alcohol (units consumed per week)	10.1 (8.1)	9.8 (6.9)	11.0 (8.8)

### Cannabis Use

There were no differences between dependent and non-dependent cannabis users in the type of cannabis most commonly used, with both groups primarily using high-potency skunk (Table 2). There were no differences between cannabis groups in: the frequency that each preparation of cannabis was used as a percentage of total cannabis use occasions, for skunk, *U* = 141, *p* = 0.52, herbal preparations, *U* = 132.5, *p* = 0.36, and hash, *U* = 156.5, *p* = 0.86; mean age of onset of cannabis use, *t*(34) = 6.48, *p* = 0.52; amount of cannabis (grams) used a week, *U* = 152.5, *p* = 0.77; or the number of days of cannabis use/week, *t*(34) = 1.31, *p* = 0.2.

**Table 2:** Cannabis use in the cannabis groups.

	Dependent cannabis users (*n* = 18)	Non-dependent cannabis users (*n* = 18)
	*n*(%)	*n*(%)
Type of cannabis most commonly used		
Picture A—“Skunk”	15 (83.3)	17 (94.4)
Picture B—“Hash”	0	1 (5.6)
Picture C—“Herbal”	3 (16.7)	0

	**Median (IQR)**	**Median (IQR)**

Frequency of use of each cannabis type (%)		
Picture A—“Skunk”	80.5 (23)	85.0 (28)
Picture B—“Hash”	10 (13)	7 (16)
Picture C—“Herbal”	8 (23)	0.5 (10)
Amount used a week (grams)	5 (4.75)	4 (4.63)

	**M (SD)**	**M (SD)**

Days used per week	6.5 (0.7)	6.06 (1.3)
Age started using cannabis	15.6 (2.2)	16.1 (1.9)
SDS Score***	4.3 (1.5)	0.8 (0.8)

***p < 0.001, **p < 0.01, *p < 0.05.

The mean SDS score in the dependent group was 4.3 (±1.5), significantly higher than in the non-dependent group: 0.8 [±0.8; *t*(34) = 8.60, *p* < 0.001].

### Episodic Memory and Executive Function

There were no group differences in immediate [*F*(2, 51) = 1.89, *p* = 0.16], or delayed [*F*(2, 51) = 1.39, *p* = 0.26] story recall (Table 3). There were also no group differences on measures of executive functioning: phonemic fluency [*F*(2, 51) = 2.28, *p* = 0.11], category fluency [*F*(2, 51) = 1.27, *p* = 0.29], and alcohol fluency [*F*(2, 51) = 0.48, *p* = 0.62]. The two cannabis using groups did not differ in fluency of use [*t*(34) = 0.193, *p* = 0.85].

**Table 3:** Episodic memory and executive functioning in dependent cannabis, non-dependent cannabis, and control groups.

	Dependent cannabis users (*n* = 18)	Non-dependent cannabis users (*n* = 18)	Controls (*n* = 18)
	M (SD)	M (SD)	M (SD)
Episodic memory			
Story recall—immediate	7.3 (2.5)	7.5 (2.7)	8.9 (3.1)
Story recall—delayed	6.5 (2.2)	6.3 (2.4)	7.6 (3.2)
Executive functioning			
Phonemic fluency—letter	14.8 (5.8)	12.0 (3.7)	15.1 (4.5)
Category fluency—“vegetables”	15.6 (5.1)	13.6 (4.6)	15.9 (4.6)
Category fluency—“alcohol”	20.5 (7.6)	19.3 (4.9)	21.7 (8.8)
Category fluency—“cannabis”	20.8 (7.8)	21.3 (7.7)	–

### Depression, Anxiety, and Schizotypy

**Figure 3:**
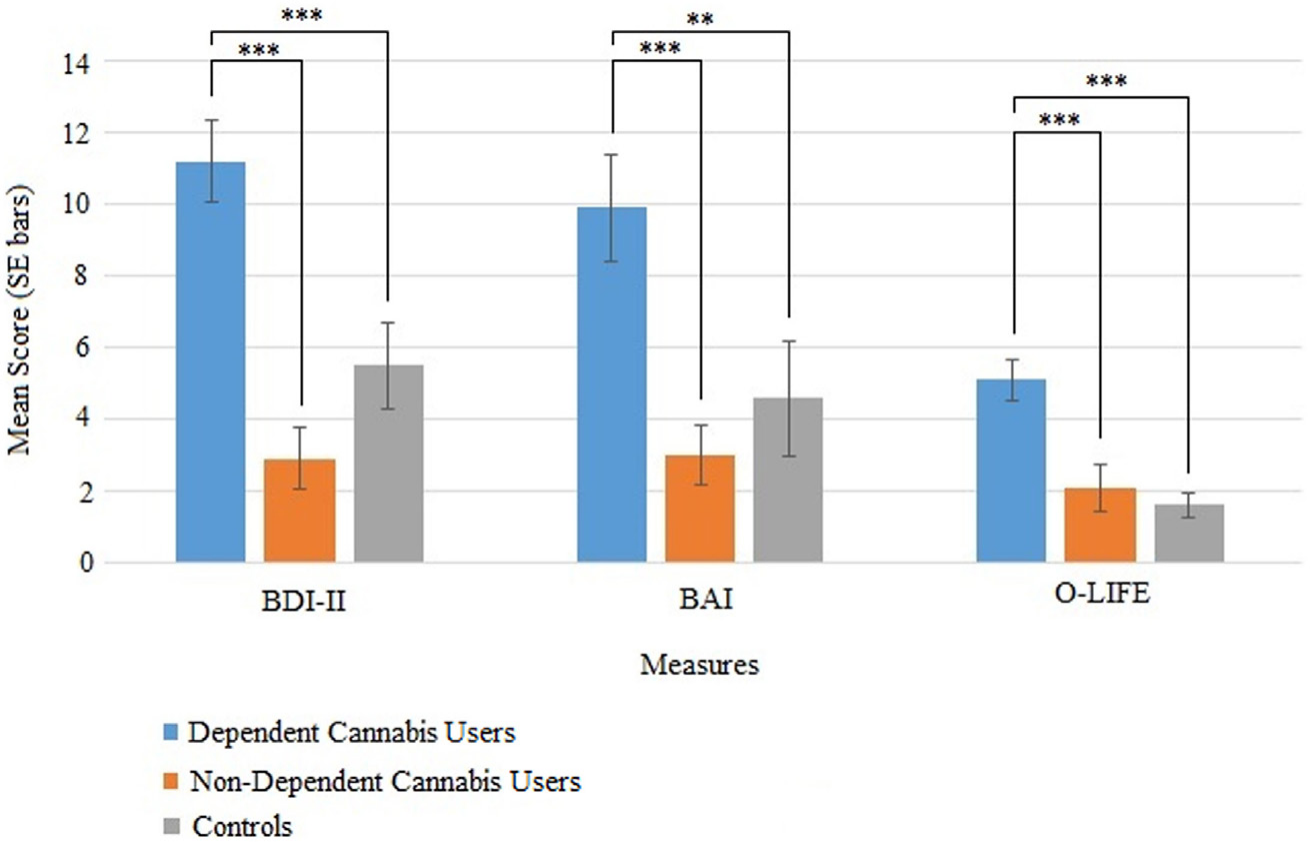
Mean (SE) scores for the BDI-II (depression), BAI (anxiety), and O-life unusual experiences (schizotypy) in the dependent cannabis, non-dependent cannabis, and control groups.

There were group differences in depression, anxiety, and schizotypy as measured by the BDI-II, *F*(2, 51) = 15.6, *p* < 0.001, BAI, *F*(2, 51) = 9.89, *p* < 0.001, and O-LIFE unusual experiences, *F*(2, 51) = 11.65, *p* < 0.001 (Table 4; Figure 3). Bonferroni-adjusted pairwise comparisons indicated that the dependent cannabis group had significantly higher levels of depression, anxiety, and schizotypy than both the non-dependent group (*p* < 0.001) and controls (*p* ≤ *p* = 0.006). The control group and non-dependent group did not significantly differ on depression (*p* = 0.28), anxiety (*p* = 1.0), or schizotypy (*p* = 1.0). According to BDI-II scores, 33% of dependent cannabis users were mildly depressed (BDI scores: 14–19), compared with 5.6% of both the non-dependent cannabis users and control group.

**Table 4:** Depression, anxiety, and schizotypy in dependent cannabis, non-dependent cannabis, and control groups.

	Dependent cannabis users (*n* = 18)		Non-dependent cannabis users (*n* = 18)		Controls (*n* = 18)	
	M (SD)		M (SD)		M (SD)	
Depression			
BDI-II total score***	11.2 (4.9)		2.9 (3.6)		5.5 (5.1)	
Anxiety			
BAI total score***	9.9 (6.3)		3.0 (3.6)		4.6 (4.5)	
Schizotypy			
O-LIFE—unusual experiences***	5.1 (2.4)		2.1 (2.8)		1.6 (1.6)	

***p < 0.001, **p < 0.01, *p < 0.05.

### Pre-FES VW

The data from the pre-FES VW days (the first virtual 2 days prior to use of FES) were first analysed to assess any baseline group differences in PM performance (Table 5). The dependent variable was the proportion of tasks that were completed correctly and analysed using a 2 × 2 × 3 repeated measures ANOVA with the within-subjects factors of task regularity (irregular, regular), task cue (event-based, time-based), and between-subjects factor of group (dependent cannabis, non-dependent cannabis, and controls). There was a significant main effect of task cue, *F*(1, 51) = 10.07, *p* = 0.003, with Bonferroni-adjusted pairwise comparisons indicating that the proportion of correct responses was greater on event-related tasks (M = 0.82, SD = 0.19) than time-related (M = 0.72, SD = 0.25).

**Table 5:** Comparison of dependent cannabis, non-dependent cannabis, and control groups on mean (SD) proportion of irregular and regular prospective memory (PM) tasks completed correctly in the prefuture event simulation virtual week.

PM task	Dependent cannabis users (*n* = 18)	Non-dependent cannabis users (*n* = 18)	Controls (*n* = 18)
			
	M (SD)	M (SD)	M (SD)
Irregular			
Event-based	0.82 (0.17)	0.86 (0.21)	0.85 (0.23)
Time-based	0.61 (0.31)	0.78 (0.21)	0.69 (0.33)
Regular			
Event-based	0.88 (0.15)	0.74 (0.25)	0.75 (0.34)
Time-based	0.76 (0.28)	0.72 (0.28)	0.72 (0.36)

There was no main effect of task regularity, *F*(1, 51) = 0.39, *p* = 0.845, or interaction between group and task cue, *F*(1, 51) = 1.07, *p* = 0.352, group and task regularity, *F*(1, 51) = 2.68, *p* = 0.078, cue and task regularity, *F*(1, 51) = 3.403, *p* = 0.071, or a three-way interaction between task regularity, task cue, and group, *F*(1, 51) = 0.093, *p* = 0.912. The outcome of a Bayesian ANOVA (BF_01_ = 4.15) was consistent with the standard analysis, indicating “moderate” evidence (35) for the null hypothesis, an absence of a three-way interaction.

### VW With FES

To assess the impact of FES on PM performance, the proportion of irregular VW tasks completed correctly before the introduction of FES was compared with the FES condition. This was done for event-based tasks and time-based tasks separately, with two 2 × 3 repeated measures ANOVAs with the within subject factors of imagining (no FES, FES) and the between-subjects factor of group (dependent cannabis, non-dependent cannabis, and controls).

For event-based irregular tasks, there was no main effect of FES, *F*(1, 51) = 1.98, *p* = 0.165, nor an interaction between FES condition (FES; no FES) and group, *F*(1, 51) = 0.258, *p* = 0.774. Similarly, for time-based irregular tasks there was no main effect of FES condition, *F*(1, 51) = 0.337, *p* = 0.564, nor an interaction between FES and group, *F*(1, 51) = 0.861, *p* = 0.429. Bayes Factor ANOVAs indicated “moderate” evidence (35) for the null hypotheses for both the event-based irregular task ANOVA (BF_01_ = 5.6) and the time-based ANOVA (BF_01_ = 3.65).

### Correlations

Exploratory analyses examined the extent of association between cognitive task performance with intellectual functioning (spot-the-word score) and amount of cannabis use (grams per week). PM (overall proportion correct on the VW), episodic memory (immediate and delayed story recall), and executive functioning (average fluency score) were correlated with spot-the-word score for all groups, and also with amount of cannabis used for the cannabis groups. Specifically, there was a strong positive correlation in non-dependent cannabis users between STW score and immediate episodic memory (*r_s_* = 0.69, *p* = 0.002). Across all three groups there were strong positive correlations between delayed episodic memory and STW score (dependent cannabis users: *r_s_* = 0.57, *p* = 0.01; non-dependent cannabis users: *r_s_* = 0.6, *p* = 0.008; controls: *r_s_* = 0.51, *p* = 0.03). No other correlations were significant.

## Discussion

### Key Findings

#### Summary

This is the first study to examine PM (regular, irregular, time-, and event-based) and the effects of FES on PM ability in dependent and non-dependent daily cannabis users compared with non-using controls. Critically, the three groups were well-matched on key variables including age, premorbid intelligence, education level, and weekly alcohol consumption. Importantly, the two cannabis using groups did not differ in any index of cannabis use (age of first use, years used, frequency of use per week, amount used per session, and type of cannabis used). However, they did differ significantly in cannabis dependence, with SDS scores in the dependent group being more than five times greater than that of the non-dependent group.

There were no differences between groups in PM ability. The use of a strategy that required participants to mentally rehearse tasks—FES—did not lead to improvements in PM scores. All groups scored similarly on measures of episodic memory and executive functioning (fluency tasks).

In terms of mental health, dependent cannabis users scored higher than non-dependent users and non-using controls on depression, with 33% falling into the category for “mild depression” on the BDI-II compared with just 6% in each of the other two groups. Dependent cannabis users also scored higher on anxiety and schizotypy than both the non-dependent and control group. We therefore replicated the findings of van der Pol et al. (3) that dependent cannabis users are more depressed and anxious than non-dependent users. It has previously been found that cannabis users score higher than non-users on schizotypy but this is the first finding that dependent cannabis users specifically have higher scores than non-dependent, suggesting previous findings may have been due to the inclusion of dependent users among the sample of users.

#### Effect of Cannabis Use on PM

Some previous research indicates that people who use cannabis perform more poorly on tasks of PM than those who do not (17–23). Our findings (across both irregular and regular PM) go against this, as we found no differences between near-daily users and controls. Indeed, two (out of six) previous PM studies with cannabis users employing objective assessments (19, 20) measuring irregular event-based PM, and one (out of five) studies measuring irregular time-based PM, also did not find differences between cannabis users and controls (19). As such, interpreting the existing literature is difficult given the broad range of definitions of “cannabis user” used for determining inclusion across studies, and differences in measures of PM and other methodological variations. However, it does appear that in our study, when carefully controlling for demographic factors within our sample of self-selecting young adults predominantly of high-educational status, frequent cannabis use does not adversely affect PM as measured by the VW task. Another explanation of our findings is that there may be an effect of cannabis use on PM, but that it was not detected in this study due to insufficient power (despite our *a priori* power calculation) or validity issues in the VW task.

It is important to consider the characteristics of the sample used in our study when interpreting the results on PM. The participants were mainly in their 20 s, relatively high functioning and well-educated, with 50–60% of each group having at least one university degree. Further, the cannabis groups did not differ in memory or executive performance relative to the control group, which is not consistent with previous studies of frequent cannabis users [for a review see Curran et al. (5)]. To test the hypothesis that our sample included a cognitively able subgroup of cannabis users for whom the cognitive effects of using cannabis are less pronounced (or non-existent), we conducted *post hoc* correlational analyses exploring whether intellectual functioning was driving the PM ability in the cannabis groups. These were not consistent with this idea. In fact, our analyses were consistent with a large meta-analysis (36) which found that years of education did not moderate the cognitive effects of cannabis.

#### Effect of FES on PM

There were no improvements in PM performance across dependent cannabis users, non-dependent cannabis users, and control participants after the introduction of FES. FES has been shown to improve some aspects of VW performance in heavy drinkers (12) healthy individuals acutely administered alcohol (16), and social drinkers but not for those dependent on alcohol (9).

As all three groups showed high levels of performance on the VW, it may be that participants were already at a ceiling level of performance leaving little room for improvement. The two cannabis groups were also more homogeneous than the controls on this task, with the controls showing higher variation (SD) across all the components of the task than the other two groups. It is also possible that FES did not benefit our participants.

According to the constructive episodic simulation hypothesis, episodic memory combines the details of past experiences (e.g., objects, people, and locations) to depict potential future events (14). It could be that the tasks on the VW have no direct relevance to participants’ episodic memories (e.g., “telephoning Bill about babysitting”), rendering imagining the potential future event a difficult task.

#### Cannabis Use and Mental Health

Cannabis use variables measured in this study did not differ between users classified as dependent and those as non-dependent. We had anticipated that dependent users would be more likely to use higher-THC type cannabis associated with more addiction risk and more pronounced memory impairment. However, 83% of dependent users and 94% of non-dependent users primarily used high-potency cannabis, which is increasingly the most readily available preparation dominating the market (5).

The finding that dependent users scored higher on schizotypy yet smoked equally potent cannabis as the non-dependent group could indicate this subgroup are affected in a different way by their cannabis use. This dependent group may have a pre-existing reactivity to cannabis that renders them more prone to experiencing psychotic-like symptoms—or, indeed, these symptoms were pre-existing and the group were more vulnerable to becoming dependent after starting cannabis use. The dependent cannabis group were also more depressed and anxious than non-dependent users, indicating that cannabis may be used to self-medicate symptoms (37) and highlighting the importance of mental health screening.

### Limitations and Future Research

We cannot generalise beyond the population sample who participated in the study—a group of young, relatively well-educated almost daily users. We cannot assume the same results would emerge in older frequent cannabis users, those with lower educational attainment, or those seeking treatment for cannabis use disorder.

Future studies aiming to draw causal links between cannabis use and PM performance, would need to be prospective, longitudinal studies of individuals before, during and after cessation of drug use. Longitudinal studies would also help to determine whether frequent cannabis use beyond young adult age would lead to PM deficits. In addition, use of ecologically valid measures of PM performance outside the laboratory using experience sampling methods would help to determine effects of differing levels of cannabis use on everyday memory functioning. Urine tests would also provide an objective measure of other drug use and whether participants refrained from drug use on the day of testing, as the present study relied on self-report.

## Conclusion

This study was the first to test daily cannabis users (both dependent and non-dependent) on various forms of PM, and compare them to non-using controls. When carefully matched on key demographic variables, including educational level and alcohol use, and when dependent and non-dependent users did not differ on any cannabis use variable, we found no evidence for a PM deficit in young adults in any group, nor evidence that FES has any effect on performance. The two cannabis groups differed significantly in cannabis dependence, and also on scores of depression, anxiety, and schizotypy.

## Ethics Statement

This study was carried out in accordance with the recommendations of the University College London (UCL) Research Ethics Committee (ethical approval number: 5402/001). All participants provided written informed consent in accordance with the Declaration of Helsinki. The protocol was approved by the University College London (UCL) Research Ethics Committee.

## Author Contributions

RB planned and developed the study protocol, tested participants, analysed data, and wrote the manuscript. SM and JW tested participants. HC and SK helped plan and develop the protocol, supervised the research project, and contributed to writing the manuscript. PR advised on the VW procedure.

## Conflict of Interest Statement

The research was conducted in absence of any commercial or financial relationships which could be construed as a potential conflict of interest.
